# 3D printing exposure and perception in radiology residency: survey results of radiology chief residents

**DOI:** 10.1186/s41205-023-00173-z

**Published:** 2023-04-27

**Authors:** David Chen, Aravinda Ganapathy, Nihil Abraham, Kaitlin M. Marquis, Grace L. Bishop, Frank J. Rybicki, Mark J. Hoegger, David H. Ballard

**Affiliations:** 1 School of Medicine, Washington University School of Medicine, St. Louis, MO USA; 2 Department of Internal Medicine, University of California-Riverside School of Medicine, Riverside, CA USA; 3 Mallinckrodt Institute of Radiology, Washington University School of Medicine, St. Louis, MO USA; 4 Department of Radiology, University of Cincinnati College of Medicine, Cincinnati, OH USA

**Keywords:** 3D printing, Radiology, Residency

## Abstract

**Rationale and objectives:**

The purpose of this study is to summarize a survey of radiology chief residents focused on 3D printing in radiology.

**Materials and methods:**

An online survey was distributed to chief residents in North American radiology residencies by subgroups of the Association of University Radiologists. The survey included a subset of questions focused on the clinical use of 3D printing and perceptions of the role of 3D printing and radiology. Respondents were asked to define the role of 3D printing at their institution and asked about the potential role of clinical 3D printing in radiology and radiology residencies.

**Results:**

152 individual responses from 90 programs were provided, with a 46% overall program response rate (n = 90/194 radiology residencies). Most programs had 3D printing at their institution (60%; n = 54/90 programs). Among the institutions that perform 3D printing, 33% (n = 18/54) have structured opportunities for resident contribution. Most residents (60%; n = 91/152 respondents) feel they would benefit from 3D printing exposure or educational material. 56% of residents (n = 84/151) believed clinical 3D printing should be centered in radiology departments. 22% of residents (n = 34/151) believed it would increase communication and improve relationships between radiology and surgery colleagues. A minority (5%; 7/151) believe 3D printing is too costly, time-consuming, or outside a radiologist’s scope of practice.

**Conclusions:**

A majority of surveyed chief residents in accredited radiology residencies believe they would benefit from exposure to 3D printing in residency. 3D printing education and integration would be a valuable addition to current radiology residency program curricula.

## Introduction

Clinical use of 3D printing anatomic models and guides from medical imaging has been supported by organized radiological societies with educational efforts by the AUR Radiology Research Alliance [1, 2] and Radiological Society of North America 3D Printing Special Interest Group [3], and successful application and integration of category III Current Procedural Codes by the American College of Radiology [4]. One such evolving area is 3D printing, which has recently been gaining significant traction in its clinical applications. Within just the last decade, an increasing number of 3D printing labs have been established, typically within large, university-affiliated teaching hospitals [5]. Clinical applications for 3D printing are broad and range from perioperative planning via procedural stimulation to medical student anatomy education, and the use of 3D printing has led to improvements in quality of trainee comprehension and patient outcomes [6].

Many of the most salient applications of 3D printing come in the perioperative setting, and as a result, select surgery programs have begun integrating 3D printing into their own medical training curricula. 3D printed organ models have been demonstrated to substantially benefit surgical skills for learners, including medical students, general surgery residents, and attending physicians [7, 8]. There is a growing body of literature documenting the effectiveness of 3D printing in residency education and training using both subjective and objective measurements [9–11]. Within the 3D printing workflow, while surgeons serve as the primary end-users, radiologists are integral to generating 3D models from medical imaging (e.g., X-ray, CT, MRI). In addition, administration for a majority of university-based 3D printing labs stems from radiology departments [1]. As such, radiology residency programs stand to benefit from introducing their trainees to 3D printing. The establishment of category III Current Procedural Terminology codes from the Centers for Medicare and Medicaid indicates a growing trend in utilization of 3D printing and has positive implications for future reimbursement [2]. As 3D printing becomes further integrated into the radiologist’s clinical duties, future residents should understand the technology behind these applications and how best to leverage them.

Each year, the American Alliance of Academic Chief Residents in Radiology, an affiliate of the Association of University Radiologists, distributes an annual survey to radiology chief residents in Accreditation Council for Graduate Medical Education (ACGME)-accredited radiology residency programs. In this, a focused subset of 3D printing related questions was included. The purpose of this study is to summarize the results of that survey and identify areas of unmet need within residency training programs and radiology education nationwide as reported by radiology chief residents.

## Materials and methods

This was an analysis of an electronic survey distributed to radiology chief residents of ACGME-accredited radiology residency programs. This survey was designated as nonhuman research by our Institutional Review Board. SurveyMonkey (Palo Alto, CA), an online survey platform, was used to generate an anonymous electronic survey with multiple choice questions to collect information about radiology residency programs and topics relevant to radiology training. A subset of these questions included questions aimed at assessing the use of 3D printing at various radiology residents’ institutions and respondents’ opinions of 3D printing’s relationship to radiology. The survey was distributed via email using a member distribution list of the Association of University Radiologist’s affiliate subgroups of chief residents, program coordinators, and program directors. Respondents were asked to identify as a chief resident of a diagnostic or interventional radiology residency program. Responses were gathered over a two-month period from March 20, 2020, through May 15, 2020, and prospective respondents were sent email reminders at 2-week intervals. As the survey was conducted during the early stages of the COVID-19 pandemic, a separate survey covering topics relating to COVID specifically was sent to the same group of chief residents and published separately [12]. There was no overlapping data between the two surveys outside of data summarizing the number of responding individuals and programs.

To differentiate responses from residency programs with multiple chief residents, survey respondents were asked to identify their residency training program. Besides this information, no additional personal details were collected to maintain anonymity and subjectivity. Respondents had the option to skip questions, which accounts for variability in the total number of respondents for each question. Survey responses were compiled, and data were summarized and analyzed at individual and program levels.

## Results

**Fig. 1:**
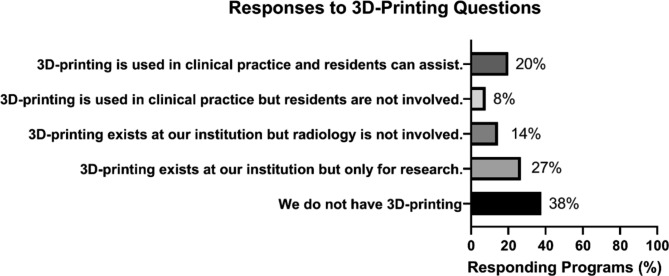
3D printing in medical practice by radiology training program. The clinical use of 3D printing by institution is indicated as a percentage of surveyed residency programs

152 radiology chief residents responded to the survey representing 90 out of 194 (46%) eligible radiology residency programs throughout North America (85 programs from the United States, 3 programs from Canada, and 2 programs from Mexico). One respondent did not answer all 3D printing questions, accounting for variability in 152 and 151 as the respondent denominator. Most respondents reported that their institutions had 3D printing (n = 54/90, 60%), with 27% (n = 24/90) indicating it is used only for research and 28% (n = 25/90) responding that it is used in clinical practice (Fig. 1). Of programs that had 3D printing, 33% (n = 18/54) allow residents to assist in the process, 13% (n = 7/54) do not involve residents, and 24% (n = 13/54) indicated that while it is available at their institution, radiology is not directly involved. Most residents (n = 91/152, 60%) feel they would benefit from some exposure to 3D printing and 6% indicated it is unlikely to be important in a radiologist’s career. When asked if 3D printing should be housed in radiology departments, 56% (n = 84/151) indicated yes, 34% (n = 52/151) were unsure, and 10% (n = 15/151) indicated no. Of the total, 33% (n = 50/151) believe it should be housed within radiology departments because of radiologists’ knowledge of imaging and segmentation, and 22% (n = 34/151) believe it would increase communication and promote a collegial relationship between radiologists and surgeons. A minority believe 3D printing is too costly, time-consuming, or outside the scope of practice for radiologists (n = 7/151, 5%), and 5% (n = 8/151) believe surgeons should have control over it.

## Discussion

This survey summarizes the role of 3D printing at institutions associated with radiology residency and radiology chief resident opinions on 3D printing’s role in radiology and if 3D printing exposure would be beneficial to radiology residency education. Although 3D printing was available at 60% of the respondents’ institutions, the degree of participation among radiologists and radiology trainees varied by institution. While most programs either do not utilize 3D printing or limit its use to research, about one-third of programs use 3D printing in clinical practice. The educational value of 3D printing in residency curricula should be considered and expanded, as most radiology chief residents believed that residents would benefit from instruction and teaching on the topic. 3D printing extends to various fields, including cardiology, neurosurgery, and obstetrics-gynecology [13–15]; education could prove beneficial to procedurally oriented residencies. Though most respondents indicated that 3D printing should be centered in radiology departments, this likely includes a bias due to sampling of only radiology residents. 3D printing will be institution-specific and ultimately determined by funding availability and financial commitment from hospital and university administrations.

Despite the exciting developments in the field of 3D printing, several factors serve to limit its overall scope and adoption in clinical settings, including significant startup costs [4] and non-uniform reimbursement [16] via category III CPT codes, which are primarily used for tracking than for robust reimbursement purposes [4]. These challenges may, in turn, temper the enthusiasm for resident participation. Specifically, since 3D printing is not yet cost–neutral from the perspective of radiology administration, there are limited situations where it will generate job vacancies in its current state. Further research in the form of formal cost analysis is required to determine whether the clinical benefits of 3D printing in the perioperative and educational settings can offset its associated benefits [17]. Despite this, 3D printing remains an exciting and potentially practice-altering technology for radiologists, and by the time reimbursement is more feasible, and funding is well established, many newly graduated radiologists will not have had sufficient training or understanding in 3D printing to take advantage of its many uses. The findings in this survey demonstrate that there is significant resident interest and enthusiasm in the integration of 3D printing training into residency curricula even in spite of reimbursement challenges and clinical adoption at a minority of hospitals. Residency programs and program directors may do well to consider integration of 3D printing training to better prepare trainees to take advantage of the clinical applications of 3D printing in their future practices.

## Limitations

This survey has limitations. This targeted radiology chief resident survey relies on respondents to identify the role of 3D printing at their institution, regardless of their knowledge or experience with 3D printing. It is possible some respondents that indicated that there was no 3D printing at their institution have 3D printing users that they were not aware of. Questions presented here asking respondents’ opinions on 3D printing and radiology are likely biased towards a response more favorable to radiology than if non-radiology trainees or attendings were also surveyed. Responses on integrating 3D printing exposure to radiology residents are influenced by individual exposure and experiences with 3D printing. As 3D printing was a subset of a more extensive survey aimed at collecting comprehensive radiology residency data, we were limited in the number of 3D printing questions we could include.

## Conclusion

The residency training period is a formative stage that allows physicians to develop competency in their clinical practice and gain exposure to their field. Radiology is a specialty defined by the intersection of technology and medicine, and radiology residency programs may consider integrating 3D printing educational material as a developing technology. A survey of radiology chief residents from accredited North American radiology programs demonstrated that a minority of radiology residencies involve 3D printing in their current curricula and clinical practices. However, most respondents are interested in learning 3D printing and incorporating it into their residency experience. Therefore, these data may be used to inform radiology educators of potential benefits and resident-level interest in integrating 3D printing education into radiology residency.

## Data Availability

Data is available by reasonable request to the corresponding author.
